# 3D Bioprinting of Polycaprolactone-Based Scaffolds for Pulp-Dentin Regeneration: Investigation of Physicochemical and Biological Behavior

**DOI:** 10.3390/polym13244442

**Published:** 2021-12-17

**Authors:** Zohre Mousavi Nejad, Ali Zamanian, Maryam Saeidifar, Hamid Reza Vanaei, Mehdi Salar Amoli

**Affiliations:** 1 Biomaterials Research Group, Department of Nanotechnology and Advance Materials, Materials and Energy Research Center, Karaj 31787-316, Iran; z.mousavinejad@merc.ac.ir (Z.M.N.); saeidifar@merc.ac.ir (M.S.); 2 Arts et Metiers Institute of Technology, CNAM, LIFSE, HESAM University, 75013 Paris, France; hamidreza.vanaei@ensam.eu; 3 OMFS-IMPATH Research Group, Department of Imaging & Pathology, Campus Sint-Rafaël, KU Leuven, Kapucijnenvoer 33, 3000 Leuven, Belgium; mehdi.salaramoli@kuleuven.be; 4 Campus Group T, Materials Technology TC, KU Leuven, Andreas Vesaliusstraat 13-Box 2600, 3000 Leuven, Belgium

**Keywords:** 3D bioprinting, tissue engineering, pulp-dentin, polycaprolactone, 45S5 Bioglass, hyaluronic acid

## Abstract

In this study, two structurally different scaffolds, a polycaprolactone (PCL)/45S5 Bioglass (BG) composite and PCL/hyaluronic acid (HyA) were fabricated by 3D printing technology and were evaluated for the regeneration of dentin and pulp tissues, respectively. Their physicochemical characterization was performed by field emission scanning electron microscopy (FESEM) equipped with energy dispersive spectroscopy (EDS), Fourier-transform infrared spectroscopy (FTIR), X-ray diffraction (XRD), atomic force microscopy (AFM), contact angle, and compressive strength tests. The results indicated that the presence of BG in the PCL/BG scaffolds promoted the mechanical properties, surface roughness, and bioactivity. Besides, a surface treatment of the PCL scaffold with HyA considerably increased the hydrophilicity of the scaffolds which led to an enhancement in cell adhesion. Furthermore, the gene expression results showed a significant increase in expression of odontogenic markers, e.g., dentin sialophosphoprotein (DSPP), osteocalcin (OCN), and dentin matrix protein 1 (DMP-1) in the presence of both PCL/BG and PCL/HyA scaffolds. Moreover, to examine the feasibility of the idea for pulp-dentin complex regeneration, a bilayer PCL/BG-PCL/HyA scaffold was successfully fabricated and characterized by FESEM. Based on these results, it can be concluded that PCL/BG and PCL/HyA scaffolds have great potential for promoting hDPSC adhesion and odontogenic differentiation.

## 1. Introduction

Tooth loss can be caused by a range of incidents and complications, including trauma, periodontal disease, or tooth decay [1,2]. Several approaches are currently used to address the problem of missing teeth, such as dentures, dental bridges, or dental implants, all of which are nonbiological methods and entail further complications. As tooth decay is one of the most common causes of tooth loss, the effective treatment of pulp necrosis has been the focus of various treatment strategies. With root canal therapy being the most widely used treatment option, the following consequences, such as brittleness of the teeth, have given rise to research on the regeneration of dental pulp as a promising alternative [3]. Tissue engineering is an approach that combines support materials and cells aimed at the regeneration of different tissues [4,5,6]. In this strategy, scaffolds provide mechanical support along with biological cues required for cells to form the new tissues, hence, playing a central part in the regeneration strategy [6,7,8]. Due to the limitations of current regenerative endodontic treatments, such as variability in the outcome, various studies have focused on the development of tooth tissue engineering scaffolds, supporting the viability and growth of cells in dental pulp and dentin, encouraging them to regenerate damaged tissue [9]. A range of techniques has been used traditionally to manufacture scaffolds for tissue engineering, including salt leaching [10], solvent casting [11], gas foaming [12], freeze casting [13], freeze drying [14], and electrospinning [15,16]. However, there are limitations associated with these methods, mainly, (1) restricted control over the microstructure (size, shape, spatial distribution, and interconnectivity of the pores), (2) difficulty with removing residual solvent from the final structure and, (3) inability to replicate complex structures [17]. A layer-by-layer deposition of materials, known as additive manufacturing or 3D printing, enables the production of three dimensional constructs with complex shapes in a significantly more facile manner compared to other techniques [18,19,20,21,22]. Moreover, the use of 3D printing techniques for regeneration applications of various tissues including pulp [23] and dentin [24] has been attracting increasing attention due to its promising results.

Presenting a host of opportunities, 3D-printed scaffolds made out of polymers, ceramics, or composites are being widely investigated as candidates for dental tissue engineering [24,25,26]. Polycaprolactone (PCL) is a biocompatible, biodegradable, and printable polymer with a reasonably high mechanical strength, which has been approved by the FDA to be used in medical devices [27]. Because of these favorable properties, PCL has been the most widely used material among the candidate materials [24]. However, PCL suffers from disadvantages such as being hydrophobic or lack of support for cell adhesion [7]. To address these challenges, various methods and techniques have been proposed, such as surface treatment with hyaluronic acid (HyA) [28] or supplementing with additional active materials. Based on the literature, HyA is a promising biomaterial for use in pulp-dentin regeneration due to its ability to enhance cellular metabolism leading to increased deposition of the mineralized matrix deposition by human dental pulp stem cells (hDPSCs) [29]. In addition, 45S5 Bioglass (BG) which was developed by Hench et al. for first the time in 1969 [30], is a silicate glass containing 45% SiO_2_, 24.5% CaO, 24.5% Na_2_O, and 6% P_2_O_5_, in wt% [31]. This material has a great ability to bond with host tissue which makes it a potential candidate for use in both soft and hard tissue regeneration applications [32]. In the last years, a lot of studies have dealt with the incorporation of BG particles as a reinforcement for polymeric scaffolds to improve the mechanical properties, bioactivity, and biocompatibility of the scaffolds [33,34]. For these reasons and while most of the studies focused on 3D printing of dental tissues, reconstruction of either dental pulp [35,36], or dentin [24,25], it was demonstrated that due to the intertwined nature of these tissues, a successful tissue engineering approach requires development of hybrid scaffolds supporting regeneration of both tissues simultaneously. 

In this present study, two different PCL-based scaffolds were fabricated by 3D printing technique and were evaluated physicochemically and biologically by field emission scanning electron microscopy (FESEM), atomic force microscopy (AFM), water contact angle, cell viability, cell adhesion and gene expression, e.g., PCL/BG and PCL/HyA scaffolds with the aim of supporting dentin and pulp regeneration, respectively. Since pulp and dentin have a close relationship during the life of the tooth, an ideal scaffold for successful tooth tissue engineering is a bilayer scaffold where each layer differs in the geometry and material. To examine the feasibility of this idea, a novel biphasic 3D-printed scaffold was designed and successfully fabricated.

## 2. Materials and Methods

### 2.1. Preparation of BG Powder

To produce 25 g of BG powder, briefly, 41.9 mL tetraethyl orthosilicate (TEOS, Si(OC_2_H_5_)_4_; Merck, Darmstadt, Germany) was added to 62.4 mL nitric acid (1M) in a glass beaker. The mixture was stirred for 1 h to complete the hydrolysis process. Then, 3.6 mL triethyl phosphate (TEP, (C_2_H_5_)_3_PO_4_; Merck, Darmstadt, Germany), 25.2 g calcium nitrate tetrahydrate (Ca(NO_3_)_2_·4H_2_O; Merck, Germany) and 16.9 g sodium nitrate (NaNO_3_; Merck, Darmstadt, Germany) were sequentially added to the stirring mixture at 45 min intervals. The prepared sol was stored in a sealed container at room temperature for three days, followed by aging at 70 °C for one day and drying at 120 °C for one day. Eventually, the dried gel was stabilized in a furnace in a planetary ball mill at 300 rpm using a ball-to-powder mass ratio of 5 and a milling time of 1 h. Yttria-stabilized zirconia vial and 3 mm balls were utilized. The 45S5 powder was prepared for use in the PCL/BG scaffold fabrication process by sieving on sieve No. 270 (53 μm).

### 2.2. Fabrication of 3D-Printed PCL, PCL/HyA, and PCL/BG Scaffolds

**Figure 1:**
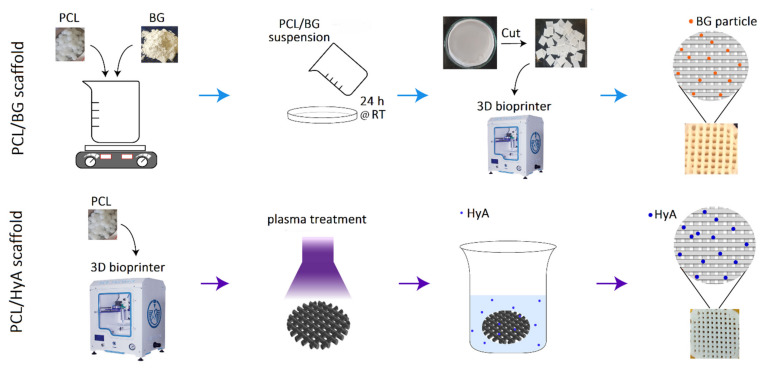
Schematic diagram of 3D-printed PCL/BG and PCL/HyA scaffold fabrication as artificial matrices for dentin and pulp tissue engineering, respectively. Abbreviations used in this Figure are as follows: PCL: polycaprolactone, BG: 45S5 bioactive glass, HyA: hyaluronic acid, h: hour, RT: room temperature.

Figure 1 delineates the method used to fabricate 3D-printed PCL, PCL/HyA, and PCL/BG scaffolds. Initially, the PCL/BG composite film was fabricated by making 5% (*w*/*v*) PCL solution (Merck, Darmstadt, Germany) in DCM (Dichloromethane; Merck, Darmstadt, Germany) on a magnetic stirrer for 3 h at 40 °C. Next, the required amount of BG to make a PCL:BG ratio of 70:30 was gradually added to the PCL solution, and the mixture was left under stirring for 1 h until a milky-white-colored suspension was obtained. Subsequently, to obtain dry films, the suspension was cast into glass petri dishes and placed in a clean environment at room temperature. To print the scaffolds, dried films were cut into 5 mm slices and loaded into 3D printer (3D BIOPRINTER N2, 3DPL Co. Ltd., Tehran, Iran) cartridges at a temperature of 90 °C, pressure 6 bar, and speed of 2 mm/s. The pure PCL film was prepared through the same protocol by casting PCL solution. The printing process was the same as PCL/BG scaffolds. To fabricate PCL/HyA scaffolds, a two-stage technology was used, consisting of plasma treatment of pure PCL scaffolds (LFG 40, Diener Electronic, Ebhausen, Germany) and subsequent immobilization of HyA on its surface. PCL scaffolds were placed in the chamber (frequency of 40 kHz, power of 100 W, and pressures of 0.6 mbar), and both top and bottom sides were exposed to plasma for 5 min (total exposure time = 10 min). The aim of plasma treatment in this study was to activate the PCL scaffold surface before immersion in HyA solution. To coat the activated scaffolds with HyA, first 4 mg/mL HyA (1.2 MDa, bloomage Freda Biopharm Co., Ltd., Jinan, China) solution in distilled water was prepared and stored at 4 °C for 24 h. Then, plasma-treated scaffolds were immersed in HyA solution on a stirrer for 12 h. Finally, the scaffolds were freeze-dried (FD-10, Pishtaz Engineering Co., Tehran, Iran) at a temperature of −58 °C and pressure 0.5 Torr for 24 h.

### 2.3. Characterization of 45S5 Bioglass Powder

The microstructure and apatite formation ability of the BG powder was characterized using a TESCAN MIRA3 Field emission scanning electron microscopy (FESEM) equipped with energy dispersive spectroscopy (EDS). Additionally, Fourier-transform infrared spectroscopy (FTIR) was performed before and after immersion in SBF. According to the in vitro standard described by Kokubo et al., 1 g of BG powder was immersed in 20 mL SBF (in a 50 mL falcon tube) and kept in a humidified 37 °C/5%CO_2_ incubator for 14 days [37]. SBF solution was refreshed twice a week, simulating the circulation of biological fluids inside a human body. After 14 days, the sample was transferred to a glass plate and allowed to be dried at 40 °C for 12 h. Samples were coated with gold and analyzed at an accelerating voltage of 15 kV. The heat treated 45S5 powder was characterized by X-ray diffraction (XRD), using a Philips PW 3710 X-rays diffractometer equipped with CuK_α_ radiation, (λ = 1.5405 Å) operating at 40 kV and 30 mA. The chemical composition of the synthesized BG powder was analyzed by XRF using Philips PW 1480 XRF Spectrometer. Thermogravimetric analysis (TGA)-differential thermal analysis (DTA) was undertaken from 50 °C to 900 °C using TGA instrument (STA 504, TA Instruments) at a heating rate of 10 °C min^–1^.

### 2.4. Characterization of Scaffolds

FESEM/EDS were used to analyze the surface morphology and chemical composition of the samples. All the samples were sputter coated with gold for 150 s. Hydrophilicity was evaluated using a contact angle measuring (CAM) device. Images were taken using DFK 23U618 USB 3.0 Color Industrial Camera using a 2X lens. Briefly, a 4 µL droplet of distilled water was deposited at the center of the PCL, PCL/HyA, and PCL/BG scaffolds. The contact angle was measured 1 min after deposition through image processing. Three samples were analyzed for each group. 

To study the in vitro bioactivity of the samples, they were immersed in simulated body fluid (SBF) solution and then placed inside an incubator at 37 °C for 14 days. The formation of apatite crystals on the surface of the 3D-printed scaffolds was examined by FESEM/EDS. To evaluate the mechanical properties of the scaffolds, universal compressive strength test system (STM20, Santam Engineering Design Co., Tehran, Iran). Electromechanical compression testing machine equipped with a 5 kN load cell at a compression rate of 1 mm/min was used. Three replicates were tested for each sample. 

To further evaluate the impact of HyA grafting on the surface structure of PCL scaffolds, the surface topography and roughness of PCL and PCL/HyA scaffolds were examined and compared using atomic force microscopy (AFM, AP 010, Park Scientific Instruments, Suwon, South Korea). AFM images were obtained by scanning the surface in contact mode (scan rate 0.1 Hz). To compare topologies of each surface, the arithmetical mean roughness, Ra values, were determined in three random areas per sample.

### 2.5. In Vitro Cell Viability Assay

The cytotoxicity and cell viability analyses were performed on PCL/BG and PCL/HyA scaffolds using MTT (3-(4,5-dimethylthiazol-2-yl)-2,5-diphenyltetrazolium bromide) assay. Initially, 3D-printed scaffolds were cut in dimensions of 5 mm × 5 mm. The sterilized scaffolds were placed in 96-well culture plates and incubated in a 5% CO_2_ incubator at 37 °C in cell culture medium in triplicate. After 1 day, a cell suspensions of human gingival fibroblast (HGF) cells (Pasteur Institute of Iran, Tehran, Iran) containing the cell density of 5 × 10^4^ cells/well were added in each well and left undisturbed for 24 h. Afterwards, 100 μL of MTT solution (5 mg/mL) was added to the wells to be incubated. After 4 h of incubation, the supernatant was removed carefully, 100 μL DMSO was added to each well, and the optical density was measured using an automatic microplate reader (BIO-TEK, VT, USA) at a wavelength of 570 nm. The wells without 3D-printed scaffolds were applied as control. The cell viability was calculated using the formula below [38,39]:

Cell viability (%) = [mean OD of test group/mean OD of control group] × 100

### 2.6. Cell Adhesion Assay

To assess the cell adhesion on 3D-printed PCL/BG and PCL/HyA scaffolds (disc with diameter 5 mm and thickness of 2 mm), hDPSCs (Pasteur Institute of Iran, Tehran, Iran) were seeded on the scaffolds with a density of 5 × 10^4^ cells/well and incubated in a CO_2_ incubator at 37 °C for 2 days. Following incubation, the cell-cultured samples were extracted and rinsed with PBS solution to remove non-attached cells. The cells were fixed using 4% paraformaldehyde followed by dehydration with ethanol solutions of ascending concentrations.

### 2.7. Gene Expression Analysis

After 21 days of hDPSCs culture on 3D-printed PCL/BG and PCL/HyA scaffolds (disc with diameter 5 mm and thickness of 2 mm with pore size of 200 ± 5 µm and 300 ± 5 µm for PCL/HyA and PCL/BG, respectively) with a density of 5 × 10^5^ cells/ well, the total RNA was extracted from samples using a Qiagen RNeasy Mini kit (Qiagen, Seoul, South Korea), and then, converted to complementary DNA (cDNA) with a first-strand cDNA using the TaKaRa RNA PCR Kit (AMV) Ver.3.0 (Takara Bio., San Jose, CA, USA). The differentiation of hDPSCs was monitored by measuring mRNA expression levels of differentiation markers, including osteocalcin (OCN), dentin matrix protein 1 (DMP1), and dentin sialophosphoprotein (DSPP). The selected housekeeping gene was β-actin for all real-time polymerase chain reaction (PCR) runs. 

Primer sequences for OCN, DMP 1, DSPP, and β-actin were designed based on published cDNA sequences (Table 1). The cells were cultured for a total of 3 weeks, with the differentiation medium being changed every 3–4 days. Each measurement was assessed in triplicate.

### 2.8. Statistical Analysis

All data were expressed as means ± standard deviation and represented at least three independent experiments. All data were analyzed using a two-way ANOVA test. *p*-values < 0.05 were considered significant. All analyses were carried out using GraphPad Prism version 9.0 for Windows (GraphPad Software, San Diego, CA, USA).

## 3. Results and Discussion

### 3.1. Characterization of 45S5 Bioglass Powder

**Figure 2:**
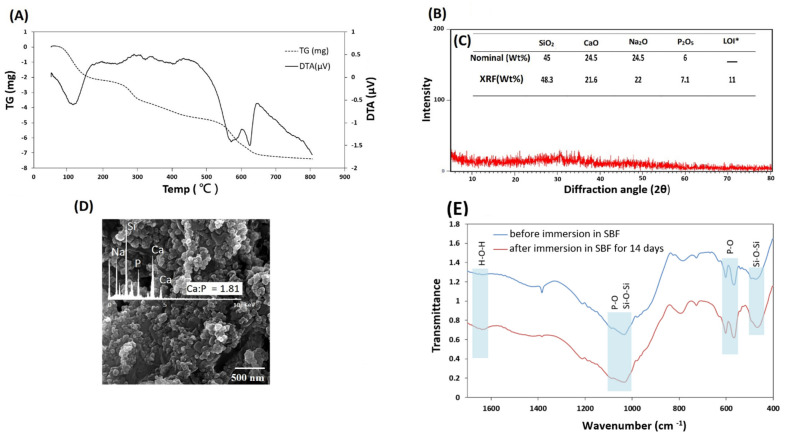
(**A**) DTA-TGA thermogram of 45S5 powder; showing a three-stage mass loss in TG and two exothermic and endothermic peaks in DTA curve. (**B**) XRD pattern of heat-treated BG; demonstrating amorphous structure. (**C**) Elemental analysis of 45S5 powder revealing similarity of the weight percentages to standard weight percentages of 45S5. * LOI: loss on ignition. (**D**) FESEM micrograph and EDS spectrum of synthesized 45S5 Bioglass powder after immersion in SBF for 14 days. (**E**) FTIR spectra of 45S5 Bioglass powder before and after immersion in SBF for 14 days. Abbreviations used in this Figure are as follows: Ca: calcium, P: phosphor, SBF: simulated body fluid.

The TGA-DTA of the BG powder was carried out to obtain the right sintering temperature. As shown in Figure 2A, the mass loss occurred in three stages. The first mass loss happened between 85 °C and 165 °C, demonstrated by an endothermic peak at 114 °C in the DTA curve assigned to the elimination of physically absorbed water, which was not removed in the drying process. Another mass loss in 250–310 °C range could be attributed to the removal of chemically absorbed water. The third mass loss took place at the 530–620 °C interval was attributed to eliminating the residual nitrates and condensation of silanol groups. The TGA trace exhibited a mass stability after 630 °C, reflected by an endothermic peak caused by glass transition, followed by an exothermic peak emerging at 650 °C. These curves confirmed that the residuals could be removed before 650 °C, which is also shown in other reports [40,41,42,43]. The result from the TGA-DTA allowed us to set the temperature of 650 °C for stabilization of the sample.

The results of XRD analysis on heat treated BG powders are demonstrated in Figure 2B. The presence of a broad hump at around 30° has been known as a hallmark of amorphous materials [44]. Consequently, this feature demonstrates the amorphous nature of the synthesized Bioglass and confirms the synthesis of Bioglass powder.

The mass oxide concentrations obtained by XRF and nominal amounts are shown in Figure 2C. The weight percentage of the element oxides is consistent with standard weight percentages of 45S5 Bioglass [45,46]. The results verify 45S5 Bioglass was produced with desired weight percentages.

In order for the scaffold to bond with native tissue, there needs to be a hydroxyapatite layer at the interface. The formation of such a layer is one of the main results of using 45S5 Bioglass in the scaffolds. This happens due to a process of glass dissolution when in contact with SBF, during which the remainder of the dissolution process leads to a change in chemical composition and environment pH, causing nucleation of hydroxyapatite. As shown in Figure 2D, formation of an apatite layer was observed on the samples’ surfaces after 14 days of immersion in SBF. Presence of an apatite phase was also validated by the EDS spectrum, as shown in Figure 2D. The ratio of Ca to P ions was approximately 1.81, which is known to be a characteristic of non-stoichiometric hydroxyapatite phase [47]. FTIR spectroscopic imaging was performed on the samples to detect the hydroxyapatite signal on the surface of SBF-treated BG powder. Figure 2E shows the spectra of BG powder before and after immersion in SBF for 14 days. The P–O bending vibration peaks at 560 and 604 cm^−1^ and the P–O asymmetric stretching vibration bands between 1000 and 1150 cm^−1^ represented the hydroxyapatite layer [48,49]. The most widely used peaks to differentiate hydroxyapatite and bioactive material are the ones corresponding to bending vibration. This is because of the superimposition of P–O stretching band and the Si–O stretching band of Bioglass, while the peak corresponding to Si-O bending was observed at 400–500 cm^−1^ [50]. The magnitude of hydroxyapatite peaks at 560 and 604 cm^−1^ increased after immersion in SBF which indicates the formation of an HA layer on the surface of the BG powder immersed in SBF solution for 14 days. The spectra exhibited bands at 1030 and 470 cm^−1^ corresponding to the Si–O–Si stretch and Si–O–Si bend, respectively [50,51,52]. The appearance of the shoulder at around 1630 cm^–1^ resulted from the H-O-H bond bending vibrations attributed to the absorbed water by the hydroxyapatite layer [53].

### 3.2. Physicocheimical Characterization of Scaffolds 

#### 3.2.1. Morphology Observations

**Figure 3:**
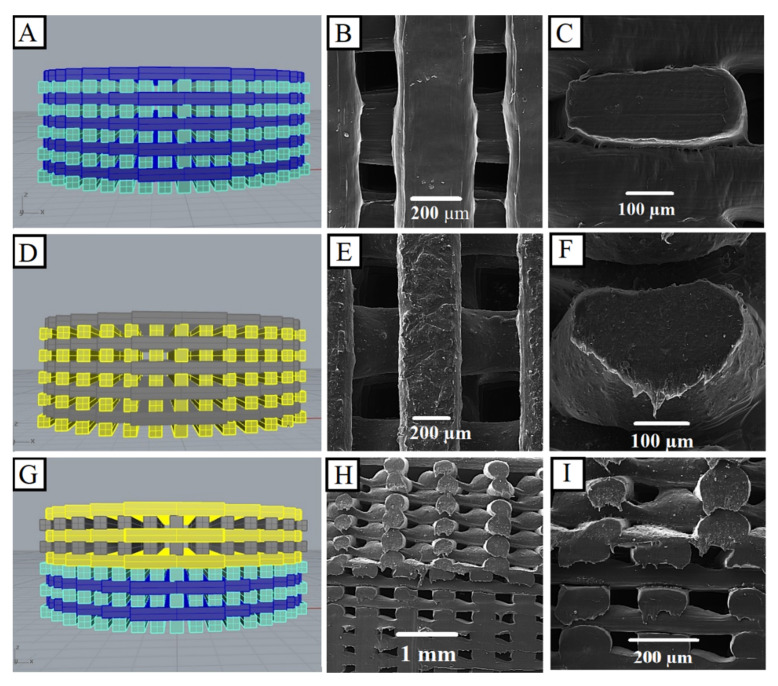
Morphological characterization of the 3D-printed PCL/BG, PCL/HyA, and bilayer scaffolds (**A**,**D**,**G**) CAD models of the scaffolds; (**B**,**E**) top view and (**C**,**F**,**H**,**I**) cross sectional FESEM images.

FESEM images of PCL/BG, PCL/HyA, and bilayer scaffolds are shown in Figure 3. As observed by FESEM, the 3D printing strategy leads to the precise production of pre-designed scaffolds (Figure 3A,D,G). The 0°/90° design was chosen for both PCL/HyA and PCL/BG scaffolds, because this pattern can be mechanically the strongest 3D-printed architecture [54]. The obtained results indicated that the scaffolds have the strut diameter of approximately 400 ± 5 µm and pore sizes of 200 ± 5 µm and 300 ± 5 µm for PCL/HyA and PCL/BG, respectively. Regarding the bilayer scaffolds, a clear transition from the PCL/HyA phase to the PCL/BG phase was observed (Figure 3H,I). FESEM observation confirmed the presence of well-distributed BG particles on both the surface and the inner part of the struts (Figure 3E,F). Furthermore, the upper and lower parts of Figure 3G–I, represent the PCL/BG phase (aimed at dentin regeneration) and the PCL/HyA phase (aimed at pulp regeneration), respectively. However, after showing the recuperation of the biological behavior of each phase, further investigation of the bilayer PCL/BG-PCL/HyA should be done in future studies.

#### 3.2.2. Atomic Force Microscopy (AFM)

**Figure 4:**
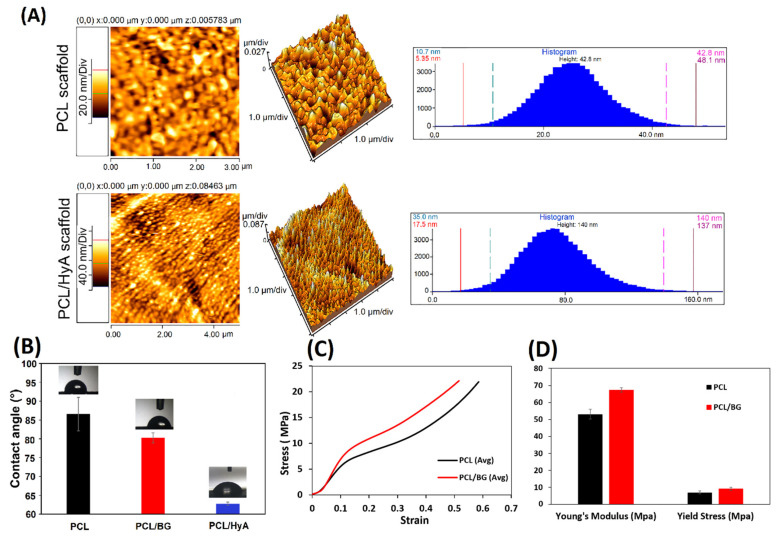
(**A**) Surface topography of 3D-printed PCL scaffolds with differential morphologies PCL and PCL/HyA obtained with AFM analysis; (**B**) contact angle measurement of 3D-printed PCL, PCL/BG, and PCL/HyA scaffolds; (**C**) compressive stress versus strain responses of PCL and PCL/BG 3D-printed scaffolds; (**D**) Young’s modulus and yield strength values of PCL and PCL/BG 3D-printed scaffolds.

To analyze and quantify the surface roughness of hyaluronic acid-grafted samples, AFM analysis was utilized (Figure 4A). A statistically significant increase in the surface roughness of the PCL/HyA scaffolds was demonstrated by AFM resulting from HyA grafting compared with PCL scaffolds (*p* < 0.01, *n* = 3). The untreated pure PCL surface showed an average roughness (R_a_) of 42.8 nm. However, after the plasma treatment and hyaluronic acid coating, the PCL/HyA surface became rougher with an R_a_ of 140 nm. These values confirmed the observations made through FESEM. It is generally recognized that an increase in roughness may drastically increase the biological response due to the higher surface/volume ratio [55,56].

#### 3.2.3. Static Water Contact Angle

PCL, PCL/BG, PCL/HyA scaffolds were subjected to contact angle measurements to evaluate the effect of the composition and surface treatment. As shown in Figure 4B, the contact angles for pure PCL, PCL/BG, and PCL/HyA scaffolds were 86 ± 2°, 80 ± 1°, and 63 ± 1°, respectively. For all samples, the contact angle values were below 90°, showing a hydrophilic tendency [57]. However, a slight decrease in the contact angle was observed after the addition of BG to the PCL, which is agreement with the findings in other studies [58,59]. The reason for this subtle change in contact angle could be that BG causes a local increase of pH when dissolved, and hydroxide ions can accelerate the cleavage of ester linkages [60]. In addition, the surface treatment of PCL scaffolds with plasma and HyA immobilization resulted a significant decrease in contact angle, that showed the plasma treatment can increase the surface wettability of the PCL-based scaffolds. Therefore, the results demonstrated that oxygen plasma treatment and HyA immobilization can affect hydrophilicity more than adding 45S5 Bioglass which probably increases cell adhesion. Similar results were obtained by Bruyas et al., who found that the addition of calcium phosphate-based materials did not significantly affect the contact angle of the PCL scaffold [58].

#### 3.2.4. Mechanical Properties of 3D-Printed Scaffolds

In order to determine the impact of 45S5 Bioglass on the structural integrity of the scaffolds, the mechanical properties of porous PCL and PCL/BG scaffolds were characterized using compression strength tests. Figure 4C demonstrates the representative compressive stress versus strain responses of PCL and PCL/BG. The sample behaves as an elastomeric or elastic-plastic solid as shown by the three regimes: (i) a linear elastic regime, (ii) a plateau of stress resulting from macropores collapsing progressively, and finally (iii) an area of densification after the pores have totally collapsed throughout the material. The Young’s modulus increased from 51.6 ± 0.62 MPa to 67.4 ± 0.54 MPa by the addition of 45S5 Bioglass to the composition (Figure 4D), which is in the range of the average value of the Young’s modulus of PCL-based 3D-printed scaffolds characterized by other researchers [61,62]. Roohani et al. also obtained the range of 19.3–49.4 MPa for Young’s modulus by adding BG to PCL [63]. The average yield stress value was 6.1 MPa and 9.16 MPa for the PCL and PCL/BG scaffolds, respectively. It can be concluded that by adding BG to a PCL-based scaffold’s composition, the mechanical strength is increased [64].

### 3.3. Cytotoxicity Assay

**Figure 5:**
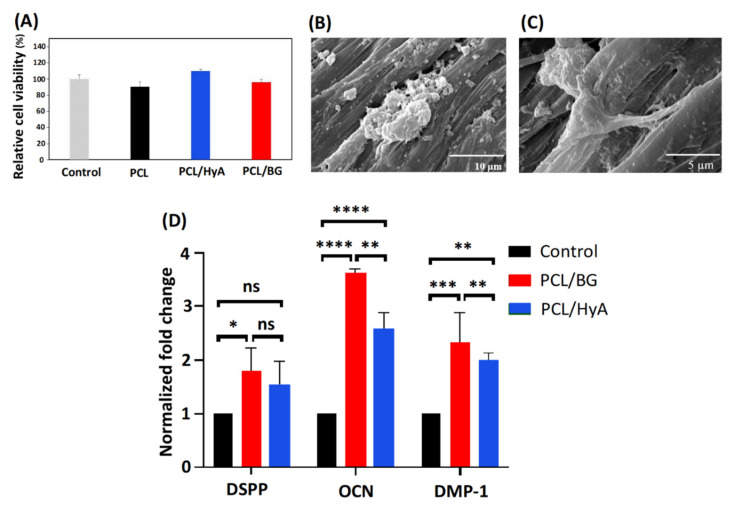
Viability of HGF cells, and morphology and differentiation ability of hDPSCs on 3D-printed scaffolds. (**A**) MTT cell viability analysis; (**B**) hDPSCs attachment on 3D-printed PCL/BG scaffold; (**C**) hDPSCs attachment on 3D-printed PCL/HyA scaffold; (**D**) Gene expression levels of DSPP, OCN, and DMP-1 in hDPSCs cultured in control conditions. ns (not significant), *p*-value > 0.05; * *p*-value ≤ 0.05; ** means *p*-value ≤ 0.01; *** means *p*-value ≤ 0.001; **** *p*-value ≤ 0.0001. Abbreviations used in this Figure are as follows: PCL: polycaprolactone, BG: 45S5 bioactive glass, HyA: hyaluronic acid, DSPP: dentin sialophosphoprotein, OCN: osteocalcin, and DMP-1: dentin matrix protein 1.

The cytotoxicity and cell viability of PCL/BG and PCL/HyA scaffolds were analyzed by MTT assay. The basis of this assay is the reduction reaction initiated by living cells’ enzymes turning a yellow MTT to purple MTT-formazan crystal [65,66]. As displayed in Figure 5A, the relative cell viability of all the samples was higher than 90%, which confirms that they are cytocompatible and suitable for use in tissue engineering applications. Hyaluronic acid-coated PCL scaffolds (PCL/HyA) presented higher cell viability than pure PCL and PCL/BG scaffolds. The lowest cell viability was found for pure PCL samples, indicating that coating PCL with a hydrophilic material like HyA or compositing with 45S5 Bioglass can improve cell adhesion by increasing the hydrophilicity of the PCL scaffolds, which is in confirmation with the result obtained by Jensen et al. [67] and Kandelousi et al. [68].

### 3.4. Cell Adhesion and Morphology Assay

Adhesion and morphology of the cells on 3D-printed PCL/BG and PCL/HyA scaffolds were analyzed using FESEM. As shown in Figure 5B,C, after two days’ culture, the hDPSCs adhered to the surface of PCL/BG and PCL/HyA scaffolds, demonstrating a uniform dispersion. The presence of long cytoplasmic prolongations on both PCL/BG and PCL/HyA scaffolds revealed an appropriate cytocompatibility of the material and positive interaction between stem cells and 3D-printed scaffolds. However, both scaffolds promoted cellular adhesion, and hDPSC felt comfortable on the surface of both PCL/BG and PCL/HyA scaffolds; it seems that PCL/Hya scaffold provides the most favorable environment for hDPSC, which is due to the effective surface modification of PCL/HyA scaffold with plasma and HyA. This finding was in agreement with the obtained results of static water contact angle. In addition, Kudryavtseva et al. [69] also proved that the surface modification with plasma and HyA enhances cell attachment. Overall, the results of this section verified the role of these modified bioactive and hydrophilic scaffolds in supporting cellular adhesion of hDPSCs and indicated the impressive potential of these scaffolds for pulp-dentin regeneration applications.

### 3.5. Gene Expression Analysis

The odontogenic differentiation ability of hDPSCs seeded on PCL/BG and PCL/HyA scaffolds as well as on a petri dish as a 2D culture was investigated after 21 days of culture in differentiation medium. The expression level of the culture dish was set to baseline (=1.0). The gene expression of dentin-associated genes including dentin sialophosphoprotein (DSPP), dentin matrix protein-1 (DMP-1), and osteocalcin (OCN) were analyzed through PCR. The results of gene expression are presented in Figure 5D with statistical differences among the groups. A very significant upregulation was observed in cells cultured on 3D-printed PCL/BG and PCL/HyA scaffolds compared to the 2D culture dish. Furthermore, the PCL/BG resulted in a significantly higher OCN and DMP-1 expression compared to PCL/HyA scaffolds (*p* < 0.01). The reason for the higher expression of DSPP, DMP-1, and OCN genes in PCL/BG scaffold compared to PCL/HyA scaffold is the presence of bioactive glass in PCL/BG, which makes the structure more mineralized and these markers are related to dentin which is a mineralized hard tissue. This difference in the expression of factors between the two scaffold types, along with the upregulation of odontoblast related genes DSPP, DMP-1 and OCN demonstrates the potential for PCL/HyA and PCL/BG scaffolds to be used in pulp and dentin regeneration, respectively, in conjunction with hDPSC.

## 4. Conclusions

With the failure of existing approaches in addressing many dental tissue complications, regenerative medicine seems like a promising approach to improve the regeneration of the dentin-pulp complex. However, traditional strategies producing scaffolds for tissue engineering in dental tissue have been ineffective. For this reason, this study presented a new strategy to fabricate 3D-printed tissue-engineering scaffolds. Polycaprolactone supplemented with 45S5 Bioglass and HyA was used to produce the scaffolds. It was shown that the coating of scaffolds with HyA had a significant impact on increasing the hydrophilicity of the scaffolds, resulting in a more favorable environment for the cells. At the same time, the addition of 45S5 Bioglass also resulted in a slightly more hydrophilic surface. The 45S5 Bioglass was found to increase the mechanical strength of the material. Furthermore, it was shown that both HyA-coated and 45S5 Bioglass-supplemented scaffolds present high cell viability. Moreover, the cellular attachment observed through FESEM and the significant upregulation of differentiation of the odontoblast-related markers DSPP, DMP-1, and OCN in both scaffold groups represent an environment assisting cellular activities. Overall, under the conditions of the present study, it might be concluded that PCL/HyA and PCL/BG scaffolds can induce an organized matrix formation similar to that of pulp and dentin tissues, respectively. The findings in this study show that the properties obtained through combining PCL with either 45S5 Bioglass or hyaluronic acid lead to the opportunity of producing a bilayer scaffold capable of assisting the regeneration of the dentin-pulp complex.

## Figures and Tables

**Table 1:** Real-time PCR primer sequences of the genes coding osteocalcin (OCN), dentin sialophosphoprotein (DSPP), and dentin matrix protein 1 (DMP1) and β-actin.

Gene	Primer Sequence	
	Forward	Reverse
OCN	5′-GCAAAGGTGCAGCCTTTGTG-3′	5′-GGCTCCCAGCCATTGATACAG-3′
DSPP	5′-CCATTCCAGTTCCTCAAAGC-3′	5′-TGGCATTTAACTCCTGTA C-3′
DMP1	5′-TTCTTTGTGAACTACGGAGG-3′	5′-TTGATACCTGGTTACTGGGA-3′
β-actin	5′-CTTCCTTCCTGGGCATG-3′	5′-GTCTTTGCGGATGTCCAC-3′

## Data Availability

Not applicable.

## References

[1] Jung C., Kim S., Sun T., Cho Y.-B., Song M. (2019). Pulp-dentin regeneration: Current approaches and challenges. J. Tissue Eng..

[2] EzEldeen M., Loos J., Mousavi Nejad Z., Cristaldi M., Murgia D., Braem A., Jacobs R. (2021). 3D-printing-assisted fabrication of chitosan scaffolds from different sources and cross-linkers for dental tissue engineering. Eur. Cell Mater..

[3] Alshehadat S.A., Thu H.A., Hamid S.S.A., Nurul A.A., Rani S.A., Ahmad A. (2016). Scaffolds for dental pulp tissue regeneration: A review. Int. Dent. Med J. Adv. Res..

[4] Shahrezaie M., Moshiri A., Shekarchi B., Oryan A., Maffulli N., Parvizi J. (2018). Effectiveness of tissue engineered three-dimensional bioactive graft on bone healing and regeneration: An in vivo study with significant clinical value. J. Tissue Eng. Regen. Med..

[5] Moshiri A., Oryan A., Shahrezaee M. (2015). An overview on bone tissue engineering and regenerative medicine: Current challenges, future directions and strategies. J. Sports Med. Doping Stud..

[6] Sohrabian M., Vaseghi M., Khaleghi H., Dehrooyeh S., Kohan M.S.A. (2021). Structural Investigation of Delicate-Geometry Fused Deposition Modeling Additive Manufacturing Scaffolds: Experiment and Analytics. J. Mater. Eng. Perform..

[7] Ghorbani F., Sahranavard M., Mousavi Nejad Z., Li D., Zamanian A., Yu B. (2020). Surface functionalization of three dimensional-printed polycaprolactone-bioactive glass scaffolds by grafting GelMA under UV irradiation. Front. Mater..

[8] Mousavi S.-M., Nejad Z.M., Hashemi S.A., Salari M., Gholami A., Ramakrishna S., Chiang W.-H., Lai C.W. (2021). Bioactive Agent-Loaded Electrospun Nanofiber Membranes for Accelerating Healing Process: A Review. Membranes.

[9] Wang F., Xie C., Ren N., Bai S., Zhao Y. (2019). Human Freeze-dried Dentin Matrix as a Biologically Active Scaffold for Tooth Tissue Engineering. J. Endod..

[10] Kanimozhi K., Basha S.K., Kaviyarasu K., SuganthaKumari V. (2019). Salt leaching synthesis, characterization and in vitro cytocompatibility of chitosan/poly (vinyl alcohol)/methylcellulose–ZnO nanocomposites scaffolds using L929 fibroblast cells. J. Nanosci. Nanotechnol..

[11] Sola A., Bertacchini J., D’Avella D., Anselmi L., Maraldi T., Marmiroli S., Messori M. (2019). Development of solvent-casting particulate leaching (SCPL) polymer scaffolds as improved three-dimensional supports to mimic the bone marrow niche. Mater. Sci. Eng. C.

[12] Rao F., Yuan Z., Li M., Yu F., Fang X., Jiang B., Wen Y., Zhang P. (2019). Expanded 3D nanofibre sponge scaffolds by gas-foaming technique enhance peripheral nerve regeneration. Artif. Cells Nanomed. Biotechnol..

[13] Pourhaghgouy M., Zamanian A., Shahrezaee M., Masouleh M.P. (2016). Physicochemical properties and bioactivity of freeze-cast chitosan nanocomposite scaffolds reinforced with bioactive glass. Mater. Sci. Eng. C.

[14] Namdarian P., Zamanian A., Asefnejad A., Saeidifar M. (2018). Evaluation of Freeze-Dry Chitosan-Gelatin Scaffolds with Olibanum Microspheres Containing Dexamethasone for Bone Tissue Engineering. Polym. Korea.

[15] Rashtchian M., Hivechi A., Bahrami S.H., Milan P.B., Simorgh S. (2020). Fabricating alginate/poly (caprolactone) nanofibers with enhanced bio-mechanical properties via cellulose nanocrystal incorporation. Carbohydr. Polym..

[16] Mirzaei Z., Kordestani S., Kuth S., Schubert D.W., Detsch R., Roether J.A., Blunk T., Boccaccini A.R. (2020). Preparation and Characterization of Electrospun Blend Fibrous Polyethylene Oxide: Polycaprolactone Scaffolds to Promote Cartilage Regeneration. Adv. Eng. Mater..

[17] Sahranavard M., Zamanian A., Ghorbani F., Shahrezaee M.H. (2019). A critical review on three dimensional-printed chitosan hydrogels for development of tissue engineering. Bioprinting.

[18] McGivern S., Boutouil H., Al-Kharusi G., Little S., Dunne N.J., Levingstone T.J. (2021). Translational application of 3D bioprinting for cartilage tissue engineering. Bioengineering.

[19] El Magri A., Vanaei S., Shirinbayan M., Vaudreuil S., Tcharkhtchi A. (2021). An Investigation to Study the Effect of Process Parameters on the Strength and Fatigue Behavior of 3D-Printed PLA-Graphene. Polymers.

[20] Vanaei H.R., Shirinbayan M., Deligant M., Khelladi S., Tcharkhtchi A. (2021). In-Process Monitoring of Temperature Evolution during Fused Filament Fabrication: A Journey from Numerical to Experimental Approaches. Thermo.

[21] Vanaei H.R., Shirinbayan M., Vanaei S., Fitoussi J., Khelladi S., Tcharkhtchi A. (2021). Multi-scale damage analysis and fatigue behavior of PLA manufactured by fused deposition modeling (FDM). Rapid Prototyp. J..

[22] Vanaei S., Parizi M.S., Vanaei S., Salemizadehparizi F., Vanaei H.R. (2021). An Overview on Materials and Techniques in 3D Bioprinting Toward Biomedical Application. Eng. Regen..

[23] Hilkens P., Bronckaers A., Ratajczak J., Gervois P., Wolfs E., Lambrichts I. (2017). The angiogenic potential of DPSCs and SCAPs in an in vivo model of dental pulp regeneration. Stem Cells Int..

[24] Wu Y., Azmi D.F., Rosa V., Fawzy A.S., Fuh J.Y., Wong Y.S., Lu W.F. (2016). Fabrication of dentin-like scaffolds through combined 3D printing and bio-mineralisation. Cogent Eng..

[25] Athirasala A., Tahayeri A., Thrivikraman G., França C.M., Monteiro N., Tran V., Ferracane J., Bertassoni L.E. (2018). A dentin-derived hydrogel bioink for 3D bioprinting of cell laden scaffolds for regenerative dentistry. Biofabrication.

[26] Nyberg E., Rindone A., Dorafshar A., Grayson W.L. (2017). Comparison of 3D-printed poly-ɛ-caprolactone scaffolds functionalized with tricalcium phosphate, hydroxyapatite, bio-oss, or decellularized bone matrix. Tissue Eng. Part A.

[27] Ghorbani F., Zamanian A., Sahranavard M. (2020). Mussel-inspired polydopamine-mediated surface modification of freeze-cast poly (ε-caprolactone) scaffolds for bone tissue engineering applications. Biomed. Eng. /Biomed. Tech..

[28] Nejad Z.M., Torabinejad B., Davachi S.M., Zamanian A., Garakani S.S., Najafi F., Nezafati N. (2019). Synthesis, physicochemical, rheological and in-vitro characterization of double-crosslinked hyaluronic acid hydrogels containing dexamethasone and PLGA/dexamethasone nanoparticles as hybrid systems for specific medical applications. Int. J. Biol. Macromol..

[29] Ahmadian E., Eftekhari A., Dizaj S.M., Sharifi S., Mokhtarpour M., Nasibova A.N., Khalilov R., Samiei M. (2019). The effect of hyaluronic acid hydrogels on dental pulp stem cells behavior. Int. J. Biol. Macromol..

[30] Hench L.L. (2006). The story of Bioglass®. J. Mater. Sci. Mater. Med..

[31] Kraxner J., Michalek M., Romero A.R., Elsayed H., Bernardo E., Boccaccini A.R., Galusek D. (2019). Porous bioactive glass microspheres prepared by flame synthesis process. Mater. Lett..

[32] Bertuola M., Aráoz B., Gilabert U., Gonzalez-Wusener A., Pérez-Recalde M., Arregui C.O., Hermida É.B. (2021). Gelatin–alginate–hyaluronic acid inks for 3D printing: Effects of bioglass addition on printability, rheology and scaffold tensile modulus. J. Mater. Sci..

[33] Singh B.N., Veeresh V., Mallick S.P., Jain Y., Sinha S., Rastogi A., Srivastava P. (2019). Design and evaluation of chitosan/chondroitin sulfate/nano-bioglass based composite scaffold for bone tissue engineering. Int. J. Biol. Macromol..

[34] Badr-Mohammadi M.-R., Hesaraki S., Zamanian A. (2014). Mechanical properties and in vitro cellular behavior of zinc-containing nano-bioactive glass doped biphasic calcium phosphate bone substitutes. J. Mater. Sci. Mater. Med..

[35] Feng S., Liu J., Ramalingam M. (2019). 3D printing of stem cell responsive ionically-crosslinked polyethylene glycol diacrylate/alginate composite hydrogels loaded with basic fibroblast growth factor for dental pulp tissue engineering: A preclinical evaluation in animal model. J. Biomater. Tissue Eng..

[36] Monteiro N., Smith E.E., Angstadt S., Zhang W., Khademhosseini A., Yelick P.C. (2016). Dental cell sheet biomimetic tooth bud model. Biomaterials.

[37] Kokubo T., Hata K., Nakamura T., Yamamuro T. (1991). Apatite formation on ceramics, metals and polymers induced by a CaO SiO2 based glass in a simulated body fluid. Bioceramics.

[38] Kılıç S., Okullu S.Ö., Kurt Ö., Sevinç H., Dündar C., Altınordu F., Türkoğlu M. (2019). Efficacy of two plant extracts against acne vulgaris: Initial results of microbiological tests and cell culture studies. J. Cosmet. Dermatol..

[39] Salehi G., Behnamghader A., Pazouki M., Houshmand B., Mozafari M. (2020). Synergistic reinforcement of glass-ionomer dental cements with silanized glass fibres. Mater. Technol..

[40] Chatzistavrou X., Zorba T., Kontonasaki E., Chrissafis K., Koidis P., Paraskevopoulos K. (2004). Following bioactive glass behavior beyond melting temperature by thermal and optical methods. Phys. Status Solidi.

[41] ElBatal H., Azooz M., Khalil E., Monem A.S., Hamdy Y. (2003). Characterization of some bioglass–ceramics. Mater. Chem. Phys..

[42] El-Ghannam A., Hamazawy E., Yehia A. (2001). Effect of thermal treatment on bioactive glass microstructure, corrosion behavior, ζ potential, and protein adsorption. J. Biomed. Mater. Res. Off. J. Soc. Biomater. Jpn. Soc. Biomater. Aust. Soc. Biomater. Korean Soc. Biomater..

[43] Lefebvre L., Chevalier J., Gremillard L., Zenati R., Thollet G., Bernache-Assolant D., Govin A. (2007). Structural transformations of bioactive glass 45S5 with thermal treatments. Acta Mater..

[44] Kumar P., Dehiya B.S., Sindhu A., Kumar V. (2018). Synthesis and Characterization of Nano Bioglass for the Application of Bone Tissue Engineering. J. Nanosci. Technol..

[45] Baino F., Hamzehlou S., Kargozar S. (2018). Bioactive glasses: Where are we and where are we going?. J. Funct. Biomater..

[46] Schnettler R., Alt V., Dingeldein E., Pfefferle H.-J., Kilian O., Meyer C., Heiss C., Wenisch S. (2003). Bone ingrowth in bFGF-coated hydroxyapatite ceramic implants. Biomaterials.

[47] Chatzistavrou X., Velamakanni S., DiRenzo K., Lefkelidou A., Fenno J.C., Kasuga T., Boccaccini A.R., Papagerakis P. (2015). Designing dental composites with bioactive and bactericidal properties. Mater. Sci. Eng. C.

[48] Ohtsuki C., Kushitani H., Kokubo T., Kotani S., Yamamuro T. (1991). Apatite formation on the surface of Ceravital-type glass-ceramic in the body. J. Biomed. Mater. Res..

[49] Pereira M.d.M., Clark A., Hench L. (1994). Calcium phosphate formation on sol-gel-derived bioactive glasses in vitro. J. Biomed. Mater. Res..

[50] Notingher I., Jones J., Verrier S., Bisson I., Embanga P., Edwards P., Polak J., Hench L. (2003). Application of FTIR and Raman spectroscopy to characterisation of bioactive materials and living cells. J. Spectrosc..

[51] Theodorou G., Goudouri O., Kontonasaki E., Chatzistavrou X., Papadopoulou L., Kantiranis N., Paraskevopoulos K. (2011). Comparative bioactivity study of 45S5 and 58S bioglasses in organic and inorganic environment. Bioceram. Dev. Appl..

[52] Brauer D.S., Karpukhina N., O’Donnell M.D., Law R.V., Hill R.G. (2010). Fluoride-containing bioactive glasses: Effect of glass design and structure on degradation, pH and apatite formation in simulated body fluid. Acta Biomater..

[53] Felisberto M.D., Laranjeira M. (2009). Preparation and characterization of hydroxyapatite-coated iron oxide particles by spray-drying technique. An. Da Acad. Bras. De Ciências.

[54] Fernandes J., Deus A.M., Reis L., Vaz M.F., Leite M. Study of the influence of 3D printing parameters on the mechanical properties of PLA. Proceedings of the 3rd International Conference on Progress in Additive Manufacturing (Pro-AM 2018).

[55] Guarino V., Gloria A., Raucci M.G., De Santis R., Ambrosio* L. (2012). Bio-inspired composite and cell instructive platforms for bone regeneration. Int. Mater. Rev..

[56] Khiabani A.B., Ghanbari A., Yarmand B., Zamanian A., Mozafari M. (2018). Improving corrosion behavior and in vitro bioactivity of plasma electrolytic oxidized AZ91 magnesium alloy using calcium fluoride containing electrolyte. Mater. Lett..

[57] Syakur A., Sutanto H. Determination of Hydrophobic Contact Angle of Epoxy Resin Compound Silicon Rubber and Silica. Proceedings of the IOP Conference Series: Materials Science and Engineering.

[58] Bruyas A., Lou F., Stahl A.M., Gardner M., Maloney W., Goodman S., Yang Y.P. (2018). Systematic characterization of 3D-printed PCL/β-TCP scaffolds for biomedical devices and bone tissue engineering: Influence of composition and porosity. J. Mater. Res..

[59] Keivani F., Shokrollahi P., Zandi M., Irani S., Shokrolahi F., Khorasani S. (2016). Engineered electrospun poly (caprolactone)/polycaprolactone-g-hydroxyapatite nano-fibrous scaffold promotes human fibroblasts adhesion and proliferation. Mater. Sci. Eng. C.

[60] Bossard C., Granel H., Wittrant Y., Jallot É., Lao J., Vial C., Tiainen H. (2018). Polycaprolactone/bioactive glass hybrid scaffolds for bone regeneration. Biomed. Glasses.

[61] Yu H.S., Park J., Lee H.-S., Park S.A., Lee D.-W., Park K. (2018). Feasibility of polycaprolactone scaffolds fabricated by three-Dimensional printing for tissue engineering of tunica albuginea. World J. Men’s Health.

[62] Seyedsalehi A., Daneshmandi L., Barajaa M., Riordan J., Laurencin C.T. (2020). Fabrication and characterization of mechanically competent 3D printed polycaprolactone-reduced graphene oxide scaffolds. Sci. Rep..

[63] Roohani-Esfahani S., Nouri-Khorasani S., Lu Z., Appleyard R., Zreiqat H. (2011). Effects of bioactive glass nanoparticles on the mechanical and biological behavior of composite coated scaffolds. Acta Biomater..

[64] Tamjid E. (2018). Three-dimensional polycaprolactone-bioactive glass composite scaffolds: Effect of particle size and volume fraction on mechanical properties and in vitro cellular behavior. Int. J. Polym. Mater. Polym. Biomater..

[65] Anderson A.S. (2020). MTT Proliferation Assay. Proc. West Va. Acad. Sci..

[66] Kamiloglu S., Sari G., Ozdal T., Capanoglu E. (2020). Guidelines for cell viability assays. Food Front..

[67] Jensen J., Kraft D.C.E., Lysdahl H., Foldager C.B., Chen M., Kristiansen A.A., Rölfing J.H.D., Bünger C.E. (2015). Functionalization of polycaprolactone scaffolds with hyaluronic acid and β-TCP facilitates migration and osteogenic differentiation of human dental pulp stem cells in vitro. Tissue Eng. Part A.

[68] Kandelousi P.S., Rabiee S.M., Jahanshahi M., Nasiri F. (2019). The effect of bioactive glass nanoparticles on polycaprolactone/chitosan scaffold: Melting enthalpy and cell viability. J. Bioact. Compat. Polym..

[69] Kudryavtseva V., Stankevich K., Kozelskaya A., Kibler E., Zhukov Y., Malashicheva A., Golovkin A., Mishanin A., Filimonov V., Bolbasov E. (2020). Magnetron plasma mediated immobilization of hyaluronic acid for the development of functional double-sided biodegradable vascular graft. Appl. Surf. Sci..

